# Characterization of *Aster lautureanus* (Asteroideae, Asteraceae) Chloroplast Genome and Phylogenetic Insights

**DOI:** 10.1002/ece3.74009

**Published:** 2026-07-09

**Authors:** Qiaoyu Zhang, Yuan Xu, Haoyu Zhang, Xiaoqing Yuan, Xiangyun Ji, Xinyue Zhang, Yan Zhang, Shoufu Gong, Zongli Chu, Zhenzhen Cheng

**Affiliations:** ^1^ College of Horticulture Xinyang Agricultural and Forestry University Xinyang P.R. China; ^2^ College of Pharmacy Xinyang Agricultural and Forestry University Xinyang P.R. China; ^3^ College of Land Resources and Environment Jiangxi Agricultural University Nanchang P.R. China; ^4^ College of Agriculture Xinyang Agricultural and Forestry University Xinyang P.R. China

**Keywords:** *Aster lautureanus*, chloroplast genome, divergence time, phylogenetic analyses

## Abstract

The genus *Aster* represents an important group of flowering plants with complex evolutionary relationships, yet genomic resources for some species remain limited. In this study, we first sequenced and characterized the complete chloroplast genome of *Aster lautureanus* and conducted comprehensive analyses of genome structure, codon usage, repeat sequences, phylogeny, and divergence time. The chloroplast genome of *A. lautureanus* was 152,545 bp in length and exhibited a typical quadripartite structure consisting of a large single‐copy (LSC) region, a small single‐copy (SSC) region, and two inverted repeats (IRs). A total of 127 genes were annotated, including 84 protein‐coding genes, 35 tRNA genes, and eight rRNA genes. Codon usage analysis revealed a strong preference for A/U‐ending codons. Neutrality, ENC, and PR2 analyses consistently indicated that natural selection plays a dominant role in shaping codon bias. A total of 85 simple sequence repeats (SSRs) and 42 long repeats were identified, suggesting potential molecular markers for future population genetic studies. Comparative analysis showed that IR regions were conserved, with minor variations at SC/IR boundaries. Phylogenetic analysis supported the placement of *A. lautureanus* within section *Orthomeris*. Divergence time estimation indicated that diversification within *Aster* mainly occurred during the Neogene and Quaternary periods, likely influenced by climatic fluctuations during the Pleistocene. This study provides valuable genomic resources and new insights into plastome evolution, phylogenetic relationships, and evolutionary history within the genus *Aster*.

## Graphical abstract

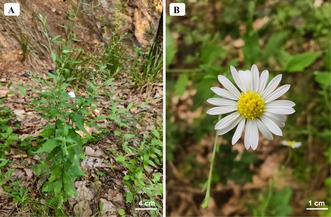

## 1 Introduction

The genus *Aster* (family Asteraceae) has been widely used for thousands of years on the Qinghai‐Tibetan Plateau for clearing of heat, detoxification, and the treatment of seasonal pandemic diseases (Li et al. 2022). *Aster lautureanus*, commonly known as the East Asian Aster, is a perennial herb native to diverse habitats, including forest margins, grasslands, and stream banks across East Asia (*Flora of China*). It is also widely used in traditional medicine for its heat‐clearing, detoxifying, and hemostatic properties based on the records of *Bencao Gangmu* (*Compendium of Materia Medica*).

Chloroplast genomes have become powerful tools in plant evolutionary biology due to their conserved structure, uniparental inheritance, and relatively slow evolutionary rate, making them particularly effective for resolving recent radiations and reconstructing phylogenetic relationships at lower taxonomic levels (Armbrust et al. 1993; Birky 2008; Parks et al. 2009; Ahmed 2015). In recent years, plastome‐scale data have significantly improved phylogenetic resolution in Asteraceae and other angiosperm lineages (Zhou et al. 2019; Wang et al. 2019; Zhang et al. 2021; Wang and Liu 2023).

Despite these advances, the evolutionary and ecological significance of chloroplast genome variation in *A. lautureanus* remains poorly understood. Therefore, a comprehensive investigation of its chloroplast genome is needed to bridge gaps between phylogeny, genome evolution, and ecological adaptation. In this study, we characterize the first complete chloroplast genome of *A. lautureanus*, perform comparative genomic analyses within Asteraceae, and reconstruct its phylogenetic position using plastome‐scale data. This work aims to provide new insights into the evolutionary history of *Aster* and to contribute to a better understanding of how chloroplast genome evolution may be associated with plant diversification in montane ecosystems.

## 2 Materials and Methods

### 2.1 Plant Collection, DNA Extraction, and Sequencing

**FIGURE 1:**
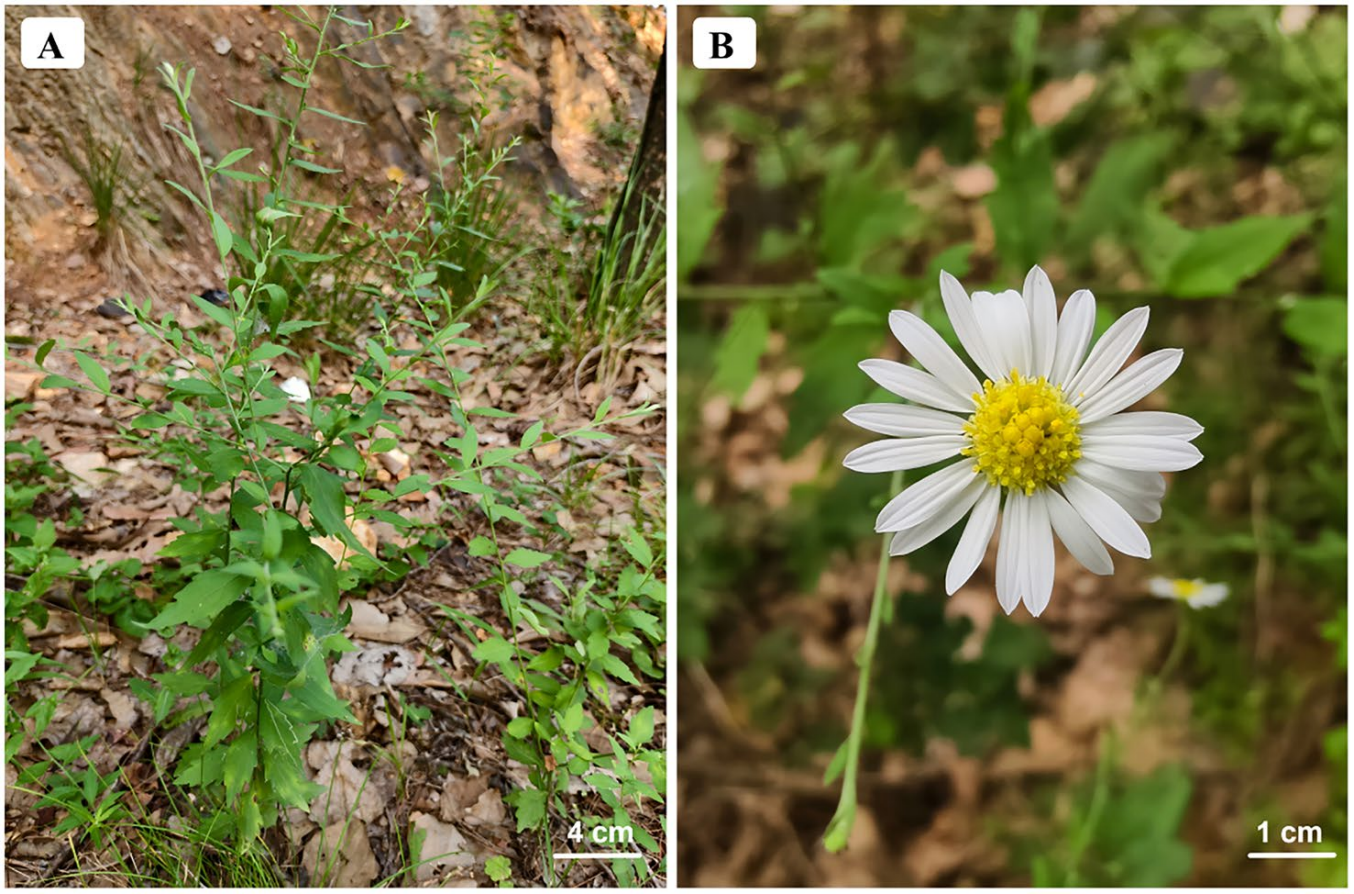
The photo of *A. lautureanus*. (A) Whole plant. (B) The flower of the plant. Photos taken by Zongli Chu in the Dabie Mountain.

A specimen of *A. lautureanus* was collected from Xinyang City, Henan Province, China (114°04′ E, 32°07′ N). The species was authenticated by Dr. Qiaoyu Zhang (Xinyang Agriculture and Forestry University, XYAFU) based on the *Flora of China*. A voucher specimen (accession number ZW20250610002; Figure 1) was deposited in the Dabie Mountain Biodiversity Herbarium.

Fresh leaves were immediately wrapped in aluminum foil and flash‐frozen in liquid nitrogen for high‐throughput sequencing. Total genomic DNA was extracted using the DNeasy Plant Mini Kit (Qiagen, Hilden, Germany) following the manufacturer's protocol. DNA quality and concentration were assessed using a NanoDrop 2000C spectrophotometer, and integrity was verified by electrophoresis on a 1% (w/v) agarose gel.

The extracted DNA was sheared to an average fragment size of ~400 bp using a Covaris M220 system (Gene Company Limited, China). A paired‐end library was constructed using the NEXTFLEX Rapid DNA‐Seq Kit (Bioo Scientific, Austin, TX, USA). Sequencing adapters containing primer hybridization sites were ligated to the fragment ends. Paired‐end sequencing was performed on the Illumina NovaSeq platform (Illumina Inc., San Diego, CA, USA) at Majorbio Bio‐Pharm Technology Co. Ltd. (Shanghai, China).

### 2.2 Genome Assembly and Annotation

Raw reads were quality‐filtered using Trimmomatic, and sequence quality was assessed with FastQC. Sequencing depth was calculated following the method described by Ni et al. (2023). Clean reads were assembled using GetOrganelle v1.7.1 with parameters “‐R 10 ‐k 21,45,65,85,105 ‐F embplant_pt” (Jin et al. 2020). Chloroplast genome annotation was performed using CPGAVAS2 (http://www.cpgavas2) (Shi, Yang, et al. 2019; Shi, Chen, et al. 2019), with *Aster flaccidus* (NC_052918.1) as the reference genome. The annotation results were manually curated in Apollo (Misra and Harris 2005). Genome structure and features were visualized using the online tool Chloroplot (Zheng et al. 2020).

### 2.3 Relative Synonymous Codon Usage (RSCU) Analysis

Codon usage patterns were analyzed using CodonW v1.3. To reduce sampling bias, only protein‐coding sequences longer than 300 bp were included in the analysis. Shorter sequences were excluded to ensure robustness. RSCU values were calculated to evaluate codon usage bias in the chloroplast genome.

### 2.4 Neutrality Plot, ENC‐Plot, and PR2‐Bias Plot Analysis

Neutrality plot analysis was conducted by plotting GC12 (the mean of GC1 and GC2) against GC3 content. A strong correlation (points close to the diagonal) indicates mutational bias as the primary driver of codon usage, whereas a scattered distribution suggests the influence of natural selection (Zhang et al. 2025). ENC‐plot analysis was performed by plotting the effective number of codons (ENC) against GC3. The expected curve was calculated using the formula:

$$\mathrm{ENC}=2+\mathrm{GC}3+29/\left[\mathrm{GC}3^{2}+{\left(1-\text{GC3}\right)}^{2}\right].$$

Deviation from the expected curve indicates the influence of natural selection over mutation (Guan et al. 2018). PR2‐bias analysis was conducted by plotting G3/(G3 + C3) against A3/(A3 + T3). The center point (A = T, G = C) represents no bias, and deviations from this point reflect directional nucleotide bias (Chaudhary et al. 2022).

### 2.5 Detection of Genomic Repetitive Sequences

Simple sequence repeats (SSRs) were identified using MISA (Beier et al. 2017), with minimum repeat thresholds set to 10, 5, 4, 3, 3, and 3 for mono‐, di‐, tri‐, tetra‐, penta‐, and hexa‐nucleotides, respectively. Long repeat sequences were detected using REPuter (Kurtz et al. 2001), considering forward, reverse, complement, and palindromic repeats.

### 2.6 Comparative Analysis of Chloroplast Genomes

The IRscope online tool (Amiryousefi et al. 2018) was used to compare junction regions among ten *Aster* species (*A. lautureanus*, *A. altaicus, A. yunnanensis, A. procerus, A. farreri, A. falcifolius, A. amellus*, *A. danyangensis*, *A. pycnophyllus*, and *A. lavandulifolius*). The analysis focused on the contraction and expansion of large single‐copy (LSC), small single‐copy (SSC), and inverted repeat (IRa and IRb) regions.

### 2.7 Phylogenetic Analysis

Phylogenetic relationships among 31 *Aster* species (Table S1) and related taxa were reconstructed using protein‐coding genes under the maximum likelihood (ML) framework, with branch support assessed based on 1000 bootstrap replicates. Two species, *Erigeron annuus* (OR909651.1) and *Erigeron breviscapus* (MK414770.1), were selected as outgroups. Sequences were aligned using MAFFT (Katoh et al. 2002). The best‐fit substitution model was selected prior to tree construction using RAxML‐NG (Minh et al. 2020). The resulting phylogenetic tree was visualized using iTOL (https://itol.embl.de/).

### 2.8 Divergence Time Estimation

Divergence times were estimated based on CDS alignments. Sequence alignment was performed using MAFFT. Three calibration points were applied: (1) *E. breviscapus*– *E. annuus* : 5.6 Mya (3.6–7.6 Mya); (2) *A. spathulifolius*– *A. tataricus* : 3.08 Mya (1.08–5.08 Mya); (3) *A. spathulifolius*–*E. breviscapus*: 8.3 Mya (5.4–14.0 Mya) (Kumar et al. 2022). Divergence time estimation was conducted using BEAST v2.7.8 under a strict molecular clock model. Markov chain Monte Carlo (MCMC) analyses were run for 50 million generations, with sampling every 1000 generations. The first 10% of samples were discarded as burn‐in. Convergence diagnostics were assessed using Tracer v1.7.2, ensuring all effective sample size (ESS) values exceeded 200. A maximum clade credibility tree was generated, and mean divergence times with 95% highest posterior density (HPD) intervals were summarized. The final tree was visualized using FigTree v1.4.4 (Bouckaert et al. 2019).

## 3 Results

### 3.1 Assembly and Characteristics of the *A. lautureanus* Chloroplast Genome

**FIGURE 2:**
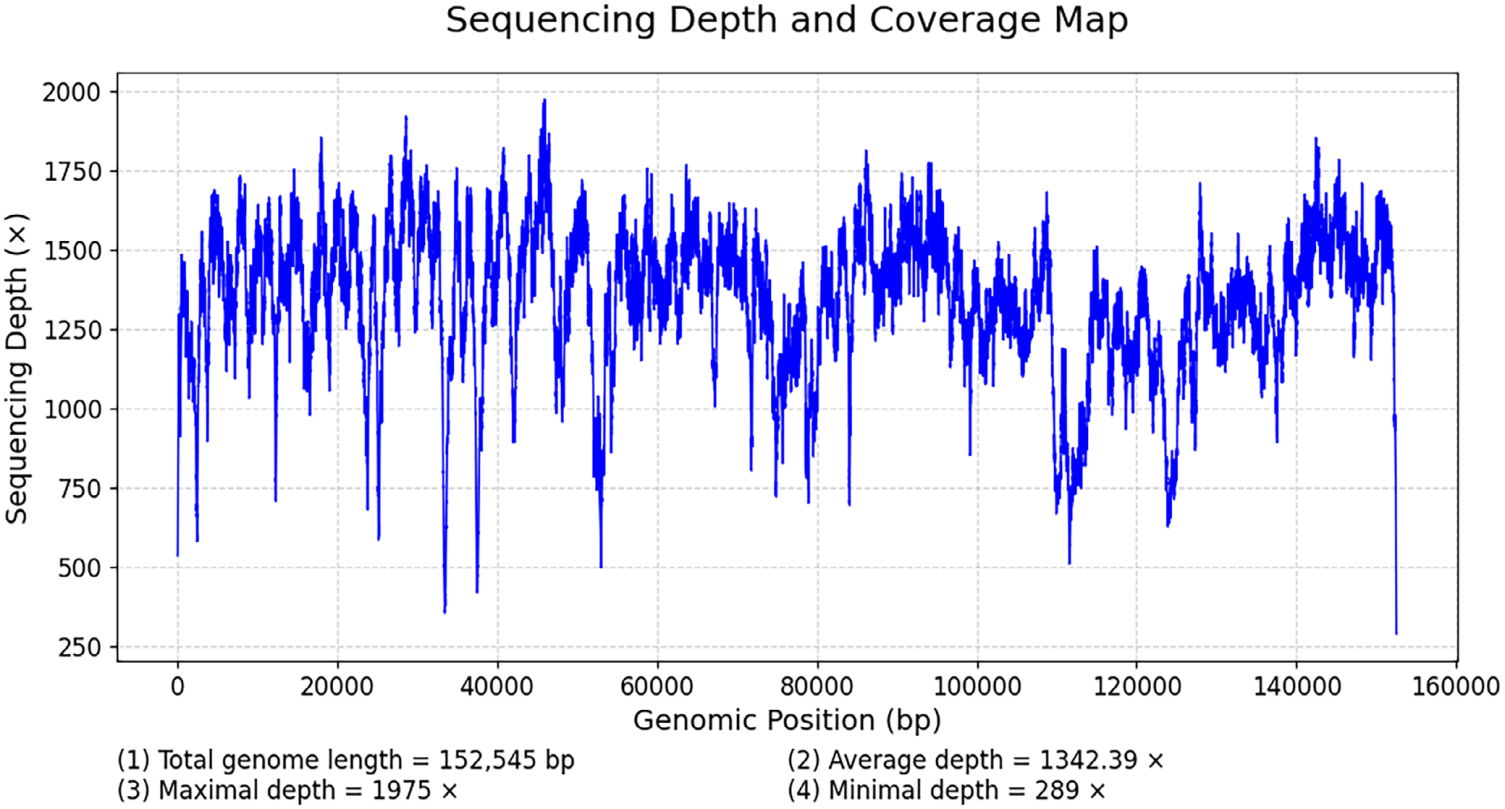
The coverage depth of the *A. lautureanus*.

Whole‐genome sequencing of *A. lautureanus* generated 8.35 Gb of raw data (SRA accession: SRR37959879), and the assembled chloroplast genome showed high sequencing depth, with an average coverage of 1342.40× (ranging from 289× to 1975×; Figure 2), indicating strong assembly reliability. The assembled genome has been submitted to GenBank under the accession number PZ250460.

**FIGURE 3:**
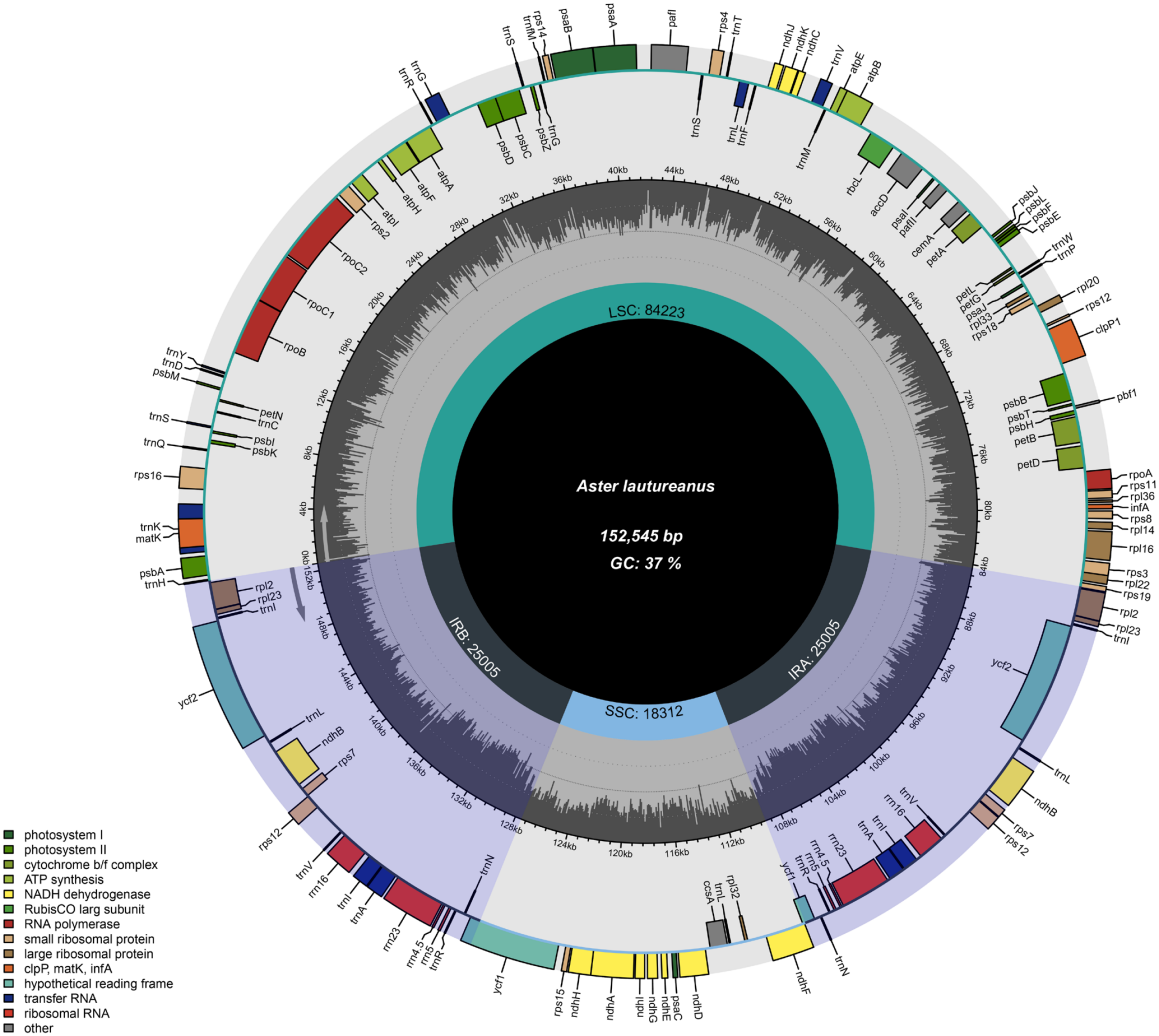
Chloroplast gene map of the *Aster lautureanus*. The outermost circle displays the gene map, with genes color coded based on their functional categories. The functional classification of the genes is shown in the bottom left corner. The middle gray inner circles represent GC content across the genome. The innermost circle depicts the genome regions: Large single‐copy (LSC), small single‐copy (SSC), and two inverted repeat regions (IRA and IRB). The species name, total size, and GC content of the genome are shown at the center of the map. The direction of the arrows indicates that the transcription directions for the inner and outer genes are clockwise and counterclockwise, respectively.

The complete chloroplast genome of *A. lautureanus* was a typical circular double‐stranded DNA molecule with a total length of 152,545 bp and an overall GC content of 37.0%. The genome exhibits the conserved quadripartite structure, consisting of a LSC region (84,223 bp), a SSC region (18,312 bp), and a pair of inverted repeat regions (IRa and IRb; 25,005 bp each) (Figure 3).

A total of 127 genes were annotated, including 84 protein‐coding genes, eight ribosomal RNA (rRNA) genes, and 35 transfer RNA (tRNA) genes. Functional classification of the protein‐coding genes revealed that 36 genes are associated with photosynthesis, 31 genes are involved in gene expression, and the remaining genes belong to other functional categories. From a broader functional perspective, 44 genes are related to photosynthesis, whereas 72 genes are involved in self‐replication processes, including rRNA and tRNA genes. In addition, six genes were assigned to other functional groups, and five genes remain functionally uncharacterized. Several genes contain introns. Specifically, *rpl16*, *petB*, *rps16*, *ndhA*, *trnI‐GAU*, and *trnA‐UGC* each contain one intron, whereas *clpP* and *ycf3* harbor two introns. Furthermore, genes located within the inverted repeat regions (e.g., *ndhB*, *rpl2*, and *trnA‐UGC*) are duplicated, and their intron lengths are identical in both copies (Table 1).

**TABLE 1:** Genetic constitution of the chloroplast genome of *A. lautureanus*.

Category	Gene group	Gene name	Quantity
Photosynthesis	Subunits of ATP synthase	atpA, atpB, atpE, atpF*, atpH, atpI	6
	Subunits of photosystem II	psbA, psbB, psbC, psbD, psbE, psbF, psbI, psbJ, psbK, psbM, psbN, psbT, psbZ, ycf3**	14
	Subunits of NADH‐dehydrogenase	ndhA*, ndhB*, ndhB*, ndhC, ndhD, ndhE, ndhF, ndhG, ndhH, ndhI, ndhJ, ndhK	12
	cytochrome b/f complex	petA, petB*, petD*, petG, petL, petN	6
	Subunits of photosystem I	*psaA, psaB, psaC, psaI, psaJ*	5
	Subunit of rubisco	*rbcL*	1
Self‐replication	Large subunit of ribosome	rpl14, rpl16*, rpl2*, rpl2*, rpl20, rpl22, rpl23, rpl23, rpl32, rpl33, rpl36	11
	DNA dependent RNA polymerase	rpoA, rpoB, rpoC1*, rpoC2	4
	Small subunit of ribosome	rps11, rps12, rps12, rps14, rps15, rps16*, rps18, rps19, rps2, rps3, rps4, rps7, rps7, rps8	14
	Ribosomal RNAs	*rrn16S、rrn23S、rrn4.5S、rrn5S、rrn5S、rrn4.5S、rrn23S、rrn16S*	8
	Transfer RNAs	trnP‐UGG、trnW‐CCA、trnM‐CAU、trnV‐UAC*、trnF‐GAA、trnL‐UAA*、trnT‐UGU、trnS‐GGA、trnfM‐CAU、trnG‐GCC、trnS‐UGA、trnG‐UCC*、trnR‐UCU、trnY‐GUA、trnD‐GUC、trnC‐GCA、trnS‐UGA、trnQ‐UUG、trnK‐UUU*、trnH‐GUG、trnI‐CAU、trnL‐CAA、trnV‐GAC、trnI‐GAU*、trnA‐UGC*、trnR‐ACG、trnN‐GUU、trnL‐UAG、trnN‐GUU、trnR‐ACG、trnA‐UGC*、trnI‐GAU*、trnV‐GAC、trnL‐CAA、trnI‐CAU	35
Other genes	Subunit of Acetyl‐CoA‐carboxylase	*accD*	1
	c‐type cytochrom synthesis gene	*ccsA*	1
	Maturase	*matK*	1
	Envelop membrane protein	*cemA*	1
	Protease	clpP**	1
	Translational initiation factor	*infA*	1
Genes of unknown function	Conserved open reading frames	*ycf1, ycf1, ycf2, ycf2, ycf4*	5
Total			127

*Note:* One asterisk = one intron; two asterisks = two introns.

### 3.2 Codon Preference Analysis

**FIGURE 4:**
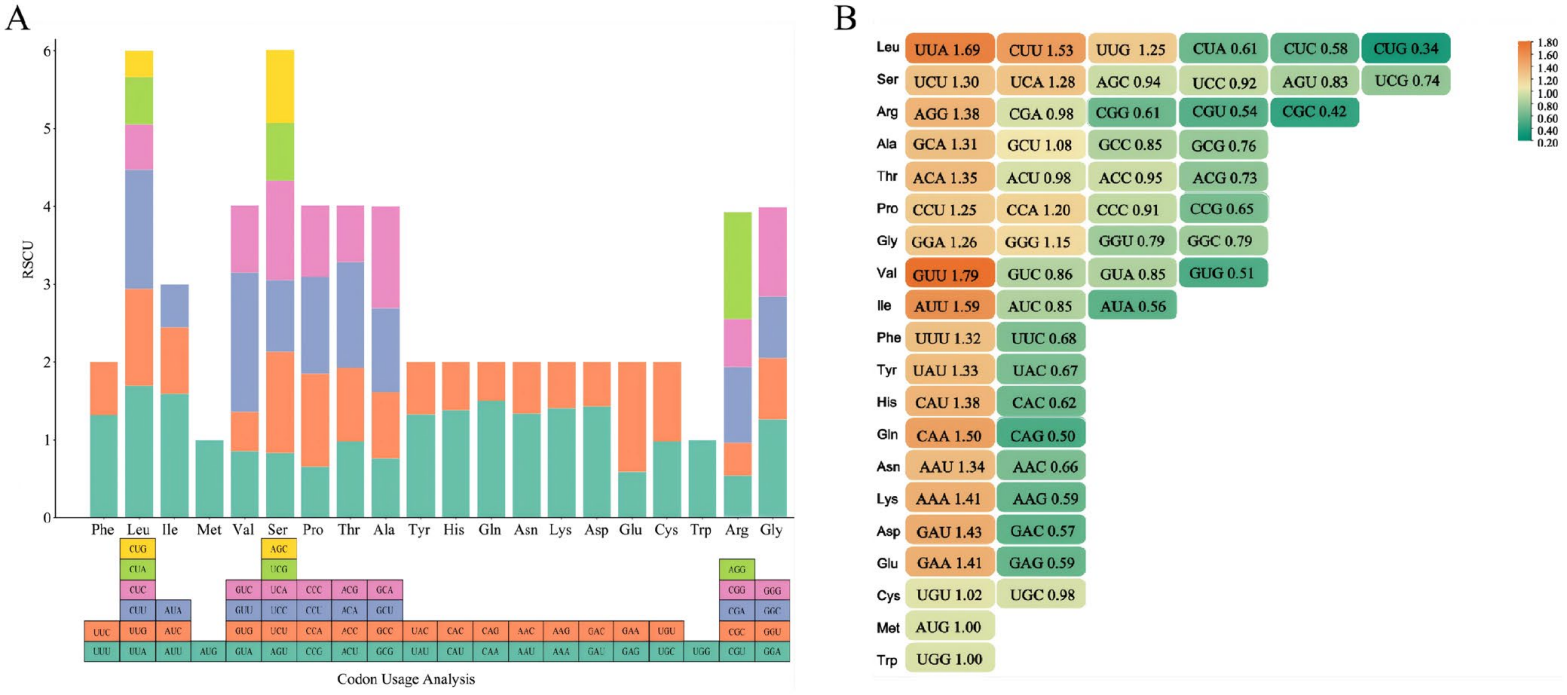
Codon usage bias analysis in the *A. lautureanus* chloroplast genome. (A) Codon content in the cp genomes. (B) Relative synonymous codon usage (RSCU) values of the cp genomes. RSCU values > 1 indicate preferential usage of a codon, while values < 1 indicate less frequent usage than expected based on equal synonymous codon frequencies.

Codon usage patterns in the chloroplast genome of *A. lautureanus* were evaluated based on RSCU values. A total of 23,942 codons encoding 20 amino acids were identified, with clear variation in codon frequencies (Figure 4A). Among all amino acids, serine (Ser) was the most abundant (2854 codons; 11.92%), whereas methionine (Met) was the least represented (253 codons; 1.06%) (Figure 4A,B). In terms of synonymous codon diversity, tryptophan (Trp) and methionine (Met) were each encoded by a single codon (UGG and AUG, respectively). In contrast, leucine (Leu) and serine (Ser) were encoded by six synonymous codons. Followed by arginine (Arg), which encoded five synonymous codons. Five amino acids—valine (Val), proline (Pro), threonine (Thr), alanine (Ala), and glycine (Gly)—were each represented by four codons, whereas isoleucine (Ile) was encoded by three codons. The remaining amino acids (Phe, Tyr, His, Gln, Asn, Lys, Asp, Glu, and Cys) were each encoded by two synonymous codons. Analysis of 63 sense codons revealed that 35 codons had RSCU values < 1, indicating relatively low usage, whereas 27 codons showed RSCU values > 1. Among the preferred codons (RSCU > 1), 24 ended with A or U, three ended with G, and none ended with C, demonstrating a strong A/U‐ending bias. The codons AUG (Met) and UGG (Trp) exhibited RSCU values of 1.00, indicating no usage bias. Among all codons, CUU (Val) showed the highest usage frequency (RSCU = 1.79), whereas UCG (Leu) was the least preferred (RSCU = 0.34) (Figure 4B).

### 3.3 Codon Usage Bias Analysis

**FIGURE 5:**
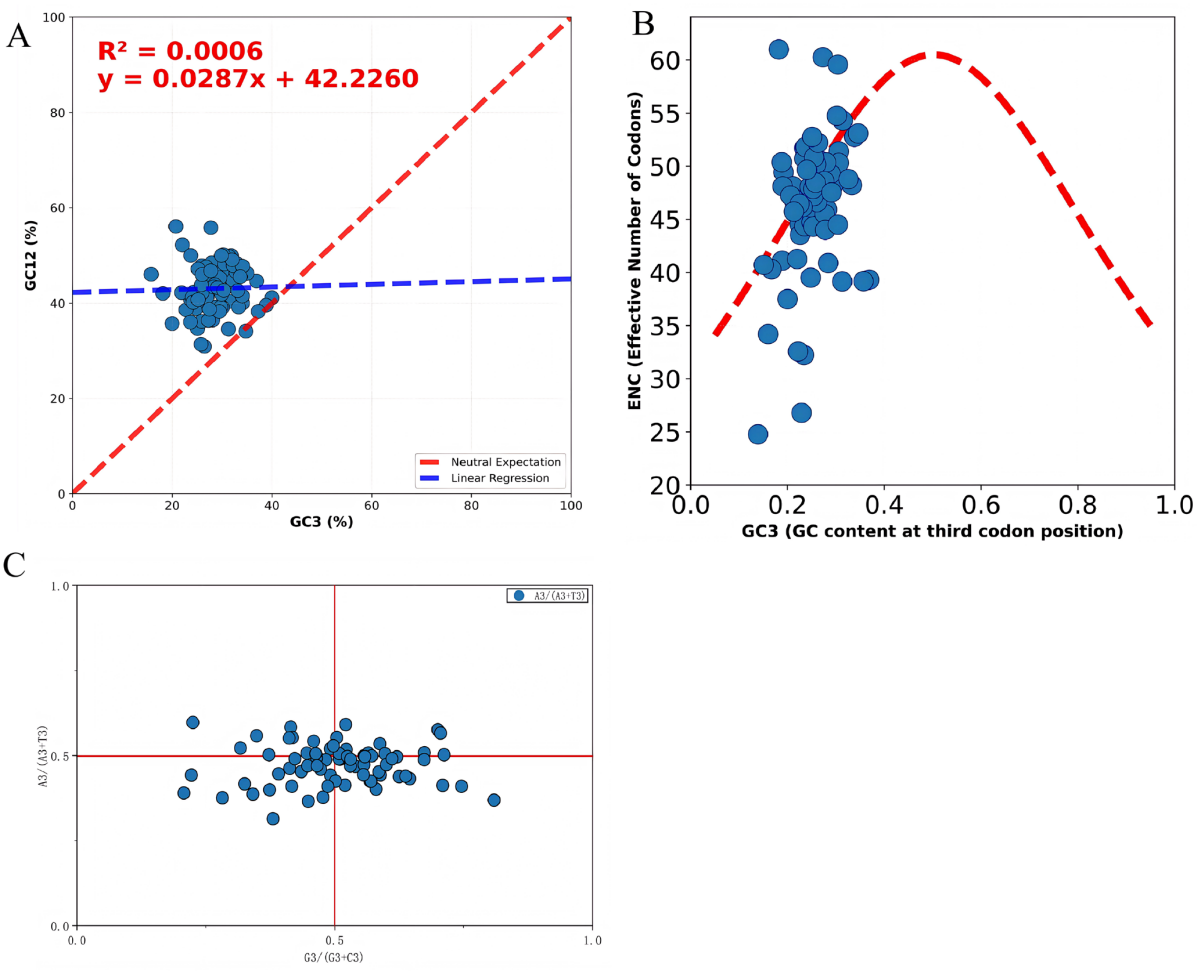
Integrated analysis of Neutrality, ENC, and PR2 bias in *A. lautureanus*. (A) Neutrality plot. (B) ENC‐GC_3s_plot. (C) PR2‐bias plot.

Neutrality plot analysis revealed that GC12 and GC3 contents ranged from 30.9% to 56.1% and from 15.8% to 40.0%, respectively (Figure 5A). The regression slope was 0.0287, far below the expected value of 1.0 under the mutation–selection equilibrium, and the coefficient of determination (*R* ^2^ = 0.0006) indicated a negligible correlation between GC12 and GC3. These results suggest that mutation pressure contributes minimally to codon usage variation, whereas natural selection plays a predominant role in shaping codon bias in the chloroplast genome of *A. lautureanus*. Consistent with this pattern, ENC–GC3 plot analysis showed that most genes fell below the expected curve (Figure 5B), indicating that codon usage deviates from neutral expectations and is therefore influenced by selective constraints rather than solely by mutational bias. PR2‐bias analysis further supported this conclusion (Figure 5C). Gene distribution was asymmetrical around the center point (0.5, 0.5), with a higher density in the lower quadrants, particularly the lower‐left region. This pattern reflects unequal usage of complementary bases at the third codon position, with a bias toward T and C over A and G. The observed imbalance indicates that both directional mutation and selective forces contribute to nucleotide composition, with natural selection exerting a stronger influence overall.

### 3.4 Expansion and Contraction of IRs

**FIGURE 6:**
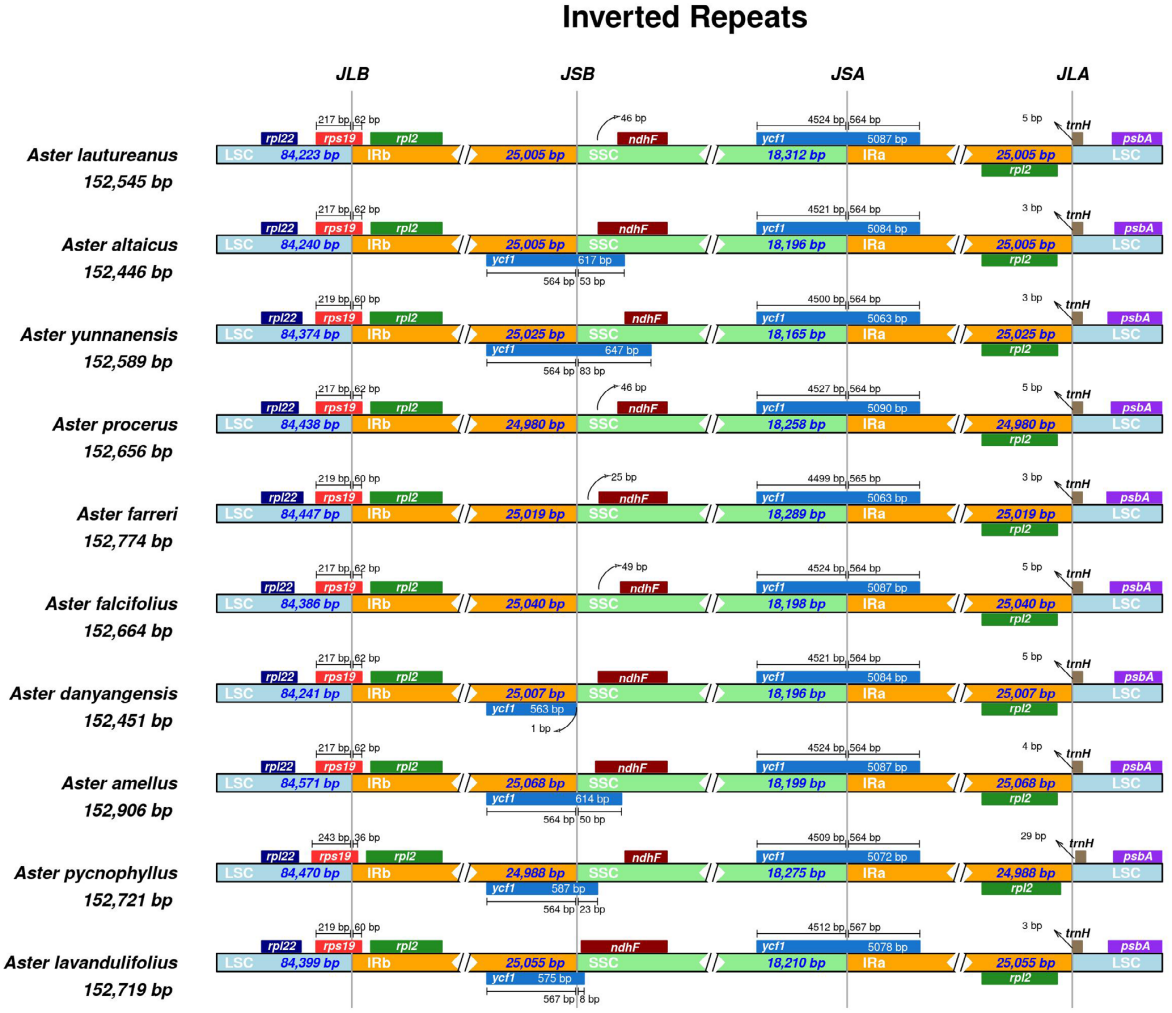
Structural comparison of inverted repeat (IR) boundaries across ten *Aster* chloroplast genomes. The diagram illustrates four key junction points: LSC/IRb (JLB), IRb/SSC (JSB), SSC/IRa (JSA), and IRa/LSC (JLA). Numbers within boxes indicate the distance (in base pairs) between gene terminals and boundary positions. Arrows demonstrate the relative positions and orientations of genes spanning these boundaries.

In general, expansion and contraction of inverted repeat (IR) regions are the primary drivers of positional variation at IR/Single‐Copy (SC) junctions in chloroplast genomes (Chen et al. 2024). We compared four IR/SC boundary regions (JLB, JSB, JSA, JLA) across 10 *Aster* chloroplast genomes to characterize junction structural divergence (Figure 6). Overall, all 10 *Aster* plastomes displayed highly conserved overall architecture, consistent gene content and collinear gene order, yet subtle length shifts and gene boundary displacements were detected at the four IR‐SC junctions.

At the JLB (LSC/IRb) boundary, the *rps19* gene spans the LSC–IRb junction in all sampled *Aster* species. The segment of *rps19* extending into the IRb region ranged from 60 bp to 62 bp in nine taxa, while *A. pycnophyllus* possessed only 36 bp, representing the most prominent contraction at JLB among all species. The IRb region consistently harbored the full *rpl2* gene across all accessions. For the JSB (IRb/SSC) junction, the *ycf1* pseudogene crossed the IRb–SSC boundary in most genomes. The portion of *ycf1* protruding from IRb into the SSC region exhibited wide interspecific variation. The photosynthesis‐related *ndhF* gene was fully localized within the SSC segment in all species, with no overlap into the IRb region. At the JSA (SSC/IRa) junction, the functional *ycf1* gene dominated the SSC–IRa boundary. The terminal fragment of *ycf1* extended into the IRa region with a nearly uniform length of 564–567 bp across all *Aster* plastomes, demonstrating strong conservation at this boundary. At JLA, the border lay between *rps12* (IRa) and *trnH* (LSC): only 3–5 bp of IRa extended toward LSC in most taxa, but 29 bp in *A. pycnophyllus*. The *trnH* and *psbA* genes were entirely retained in the LSC region for all samples, with no invasion into IRa.

### 3.5 SSRs and Long Repeat Analysis

**FIGURE 7:**
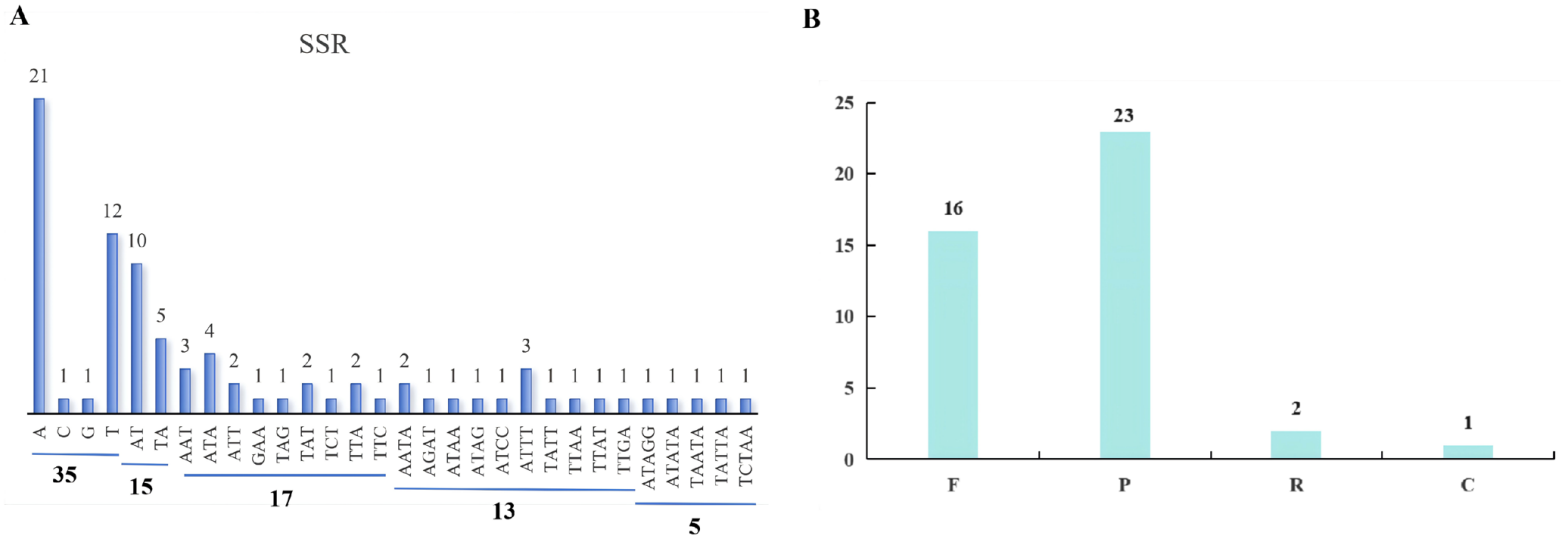
Distribution and characterization of repetitive elements in the *A. lautureanus* chloroplast genome. (A) Distribution of simple sequence repeats (SSRs) by repeat type and repeat unit size, and (B) quantitative analysis of long repeat sequences, categorized by repeat type (F, forward; P, palindromic; R, reverse; C, complementary) and length distribution.

A total of 85 SSRs were identified in the chloroplast genome of *A. lautureanus* using MISA. These SSRs were classified into five types: mononucleotide, dinucleotide, trinucleotide, tetranucleotide, and pentanucleotide repeats (Figure 7A). Mononucleotide repeats were the most abundant, accounting for 41.18% (35) of all SSRs, followed by trinucleotide (17; 20.00%), dinucleotide (15; 17.65%), tetranucleotide (13; 15.29%), and pentanucleotide repeats (5; 5.88%). No hexanucleotide repeats were detected. In terms of motif composition, mononucleotide repeats consisted primarily of A/T motifs, with a minor proportion of C/G motifs. Dinucleotide repeats were exclusively represented by the AT/AT motif. Trinucleotide repeats included three motif types: AAG/CTT, AAT/ATT, and ACT/AGT. Tetranucleotide repeats comprised five motif types (AAAT/ATTT, AATC/ATTG, AATT/AATT, AGAT/ATCT, and ATCC/ATGG), whereas pentanucleotide repeats included three motif types (AATAT/ATATT, AATCT/AGATT, and AGGAT/ATCCT). In addition to SSRs, a total of 42 long repeats (Figure 7B) were identified, including 16 forward repeats, 23 palindromic repeats, two reverse repeats, and one complementary repeat. Palindromic repeats represented the dominant type among long repeats, indicating their potential role in secondary structure formation and genome stability.

### 3.6 Phylogenetic Relationship Analysis

**FIGURE 8:**
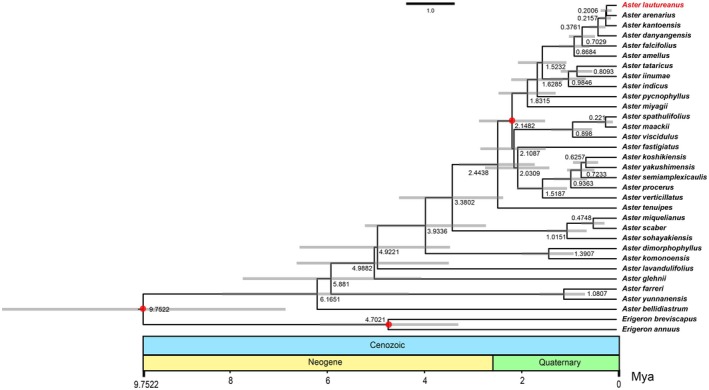
Phylogenetic relationships of *A. lautureanus* within the genus *Aster* based on CDs of plastomes using the ML method, with two *Erigerons* used as outgroups. Nodal support values are indicated as bootstrap percentages. Clade I represents the Sect. Alpigenia, Clade II represents the Sect. Aster, and Clade III represents the Sect. Orthomeris.

Phylogenetic relationships were reconstructed using complete chloroplast protein‐coding sequences from *A. lautureanus*, 30 additional *Aster* species, and two outgroup taxa ( *Erigeron annuus* and *Erigeron breviscapus*) (Figure 8). The resulting ML tree showed that *A. lautureanus* formed a well‐supported clade with *A. danyangensis*, *A. kantoensis*, *A. arenarius* , and *A. falcifolius*. These species were grouped within Clade III, which predominantly comprises members of *Aster* section *Orthomeris*.

### 3.7 Divergence Time Estimation

**FIGURE 9:**
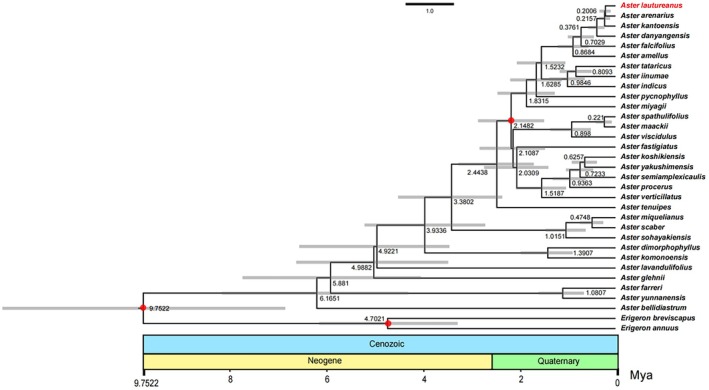
Chronogram depicting the divergence time estimated in BEAST using the CDs of chloroplast genomes. Approximately 95% posterior probability is represented by the bar on each node. Red dots at the nodes represent calibration points.

Divergence time estimation was performed based on the ML phylogenetic framework, with *Erigeron breviscapus* and *Erigeron annuus* designated as outgroups (Figure 9). The results indicated that the split between *Aster* and *Erigeron* occurred at approximately 9.75 million years ago (Mya) (95% HPD: 6.82–12.66 Mya), corresponding to the Neogene period. Within *Aster*, *A. bellidiastrum* was inferred to be the earliest diverging lineage, with an estimated divergence time of ~6.17 Mya (95% HPD: 4.28–8.12 Mya). More recent divergence events were observed among closely related species. Notably, *A. lautureanus* and *A. arenarius* diverged at approximately 0.20 Mya (95% HPD: 0.09–0.32 Mya), indicating a relatively recent speciation event. Overall, divergence within the genus *Aster* appeared to have been concentrated during the late Neogene to Quaternary periods, particularly after the onset of the Quaternary. This temporal pattern suggests that species diversification in *Aster* may have been influenced by climatic oscillations during the Pleistocene.

## 4 Discussion

### 4.1 Structural Conservation and Evolutionary Stability of the Chloroplast Genome

The chloroplast genome of *A. lautureanus* exhibits a typical quadripartite structure, consistent with most angiosperms, indicating a high level of structural conservation. The genome size, GC content, and gene composition are highly comparable to those reported in other *Aster* species (Shen et al. 2018; Wang and Liu 2023; Wang et al. 2019; Zhang et al. 2021; Zhou et al. 2019), suggesting strong evolutionary constraints on plastome organization within the genus.

The IR regions were particularly conserved, with minimal variation in length among related species (Chen et al. 2024). Such conservation is a common feature of chloroplast genomes and is thought to contribute to genome stability by reducing the rate of structural rearrangements. In contrast, slight variations in the positions of genes at SC/IR boundaries (e.g., *rps19* and *ycf1*, Figure 6) reflect minor expansion and contraction events, which are widely recognized as the primary source of plastome size variation. These findings suggest that the chloroplast genome of *A. lautureanus* has undergone limited structural divergence during evolution.

### 4.2 Codon Usage Bias Driven Primarily by Natural Selection

Codon usage analysis revealed a clear preference for A/U‐ending codons in the chloroplast genome of *A. lautureanus*, consistent with its overall AT‐rich nucleotide composition. Similar patterns have been widely observed in plant plastomes and are often associated with translational efficiency and genome composition bias (Adil et al. 2025; Liu et al. 2025; Zhang et al. 2024).

Importantly, neutrality plot, ENC‐GC3, and PR2‐bias analyses consistently indicated that natural selection plays a dominant role in shaping codon usage bias, whereas mutation pressure contributes relatively little. The extremely low correlation between GC12 and GC3, together with the deviation of most genes from the expected ENC curve, suggests that selective constraints—possibly related to gene expression efficiency, protein function, or adaptive evolution—are the primary drivers of codon preference (Song et al. 2024).

Similar results, implicating natural selection as the key driver, have been reported in plant chloroplasts (Geng et al. 2022; Yang et al. 2024), animal and plant mitochondria (Amenu et al. 2026; Uddin et al. 2016), which highlight that even in relatively conserved organellar genomes, selective forces can significantly influence molecular evolution.

### 4.3 SSRs And Long Repeats: Implications for Genome Variation and Molecular Markers

Many SSRs were identified in the chloroplast genome of *A. lautureanus*, with mononucleotide A/T repeats being the most abundant. This AT‐rich SSR distribution is consistent with the overall nucleotide composition of the genome and has been reported in many plant species (Chen et al. 2022; Parmar et al. 2022; Zhong et al. 2025).

SSRs and long repeats are known to exhibit high levels of polymorphism and are therefore valuable molecular markers for population genetics, species identification, and phylogeographic studies (Kishore et al. 2012; Yang et al. 2025). The SSRs identified in this study may provide useful resources for future investigations of genetic diversity and population structure in *Aster* species.

### 4.4 Evolutionary History and the Role of Climatic Fluctuations

Divergence time estimation suggests that the split between *Aster* and *Erigeron* occurred during the Neogene, followed by diversification within *Aster* primarily during the late Neogene and Quaternary periods. Notably, the relatively recent divergence between *A. lautureanus* and *A. arenarius* indicates ongoing or recent speciation processes within the genus.

The concentration of diversification events during the Quaternary, particularly the Pleistocene, suggests that climatic oscillations may have played a key role in shaping species evolution. Repeated cycles of glaciation and interglacial periods could have promoted geographic isolation, habitat fragmentation, and subsequent speciation. In the genus *Mikania* (Asteraceae) (de Godoy et al. 2017), based on molecular data and divergence time estimation, two major evolutionary lineages were formed approximately 6.0 Mya. The inferred Pleistocene climatic forcing of species diversification echoes the Neogene–Quaternary diversification pattern observed in the genus *Aster* (also in Asteraceae), reinforcing the climatic sensitivity of its evolutionary history.

Together, these findings indicate that both long‐term evolutionary constraints and more recent environmental changes have contributed to the diversification of *Aster* species.

## 5 Conclusion

In this study, we first characterized the complete chloroplast genome of *A. lautureanus* and performed comprehensive analyses of genome structure, codon usage, repeat sequences, phylogenetic relationships, and divergence times. The chloroplast genome exhibited a conserved quadripartite structure with limited variation, reflecting strong evolutionary stability. Codon usage bias was predominantly shaped by natural selection, with a clear preference for A/U‐ending codons. The identification of abundant SSRs and repeat elements provides valuable resources for future genetic and evolutionary studies. Phylogenetic analysis robustly placed *A. lautureanus* within section *Orthomeris*, while divergence time estimation revealed that species diversification in *Aster* was largely associated with climatic changes during the Neogene and Quaternary periods. Overall, this study provides important insights into the evolutionary history, genome organization, and molecular characteristics of *A. lautureanus*, and contributes to a better understanding of plastome evolution and phylogenetic relationships within the genus *Aster*.

## Author Contributions

**Qiaoyu Zhang:** data curation (equal), investigation (equal), methodology (equal), writing – original draft (equal), writing – review and editing (equal). **Yuan Xu:** conceptualization (equal), data curation (equal). **Haoyu Zhang:** data curation (equal), formal analysis (equal), software (equal). **Xiaoqing Yuan:** data curation (equal), formal analysis (equal), software (equal). **Xiangyun Ji:** data curation (equal), formal analysis (equal), software (equal). **Xinyue Zhang:** data curation (equal), formal analysis (equal), software (equal). **Yan Zhang:** data curation (equal), funding acquisition (equal), project administration (equal). **Shoufu Gong:** data curation (equal), funding acquisition (equal), project administration (equal). **Zongli Chu:** data curation (equal), formal analysis (equal), funding acquisition (equal), resources (equal), writing – review and editing (equal). **Zhenzhen Cheng:** data curation (equal), formal analysis (equal), funding acquisition (equal), resources (equal), writing – review and editing (equal).

## Funding

This research was supported by Science and Technology Tackling in Henan Province under (252102110251), (252102110224), (252102110233), (24210211165), and (252102110329); Key Project of the Dabie Mountains Laboratory (Grant number DMLP013).

## Ethics Statement

The sample of *A. lautureanus* was collected from Dabie Mountain, Xinyang City, Henan Province, China, and strictly complied with local and Chinese regulations. No specific permission was required for the collection of the samples used in this study.

## Conflicts of Interest

The authors declare no conflicts of interest.

## Supporting information


**Table S1:** *Aster* species and outgroup for phylogenetic tree.

## Data Availability

The genome sequence data that support the findings of this study are openly available in GenBank of NCBI at https://www.ncbi.nlm.nih.gov/ under the accession number PZ250460. The associated BioProject, BioSample, and SRA numbers are PRJNA1449060, SAMN57109248, and SRR37959879, respectively.
